# Neuromodulatory Correlates of Pupil Dilation

**DOI:** 10.3389/fncir.2018.00021

**Published:** 2018-03-09

**Authors:** Rylan S. Larsen, Jack Waters

**Affiliations:** Allen Institute for Brain Science, Seattle, WA, United States

**Keywords:** pupillometry, noradrenaline, norepinephrine, acetylcholine, neuromodulation

## Abstract

Pupillometry has long been used as a measure of brain state. Changes in pupil diameter are thought to coincide with the activity of neuromodulators, including noradrenaline and acetylcholine, producing alterations in the brain state and corresponding changes in behavior. Here we review mechanisms underlying the control of pupil diameter and how these mechanisms are correlated with changes in cortical activity and the recruitment of neuromodulatory circuits.

## Introduction

An animal's behavior and neuronal responses are modulated by changes in brain state. Brain states can be defined as periods of neuronal network activity that correlate with behaviors such as periods of arousal, locomotion, exploration, and attention (Lee and Dan, 2012). For example, high frequency fluctuations in pyramidal cell membrane potential, local field potential (LFP), and electroencephalogram (EEG) are observed in sensory cortices during attentive or activated states (Steriade, 1997; Crochet and Petersen, 2006). Similarly, sharp wave ripples are observed in hippocampus during periods of reduced activity such slow wave sleep, quiet wakefulness, and grooming (Buzsáki, 1986). Changes in network activity are often accompanied by changes in neuronal responsiveness and pupil diameter (McGinley et al., 2015). As a result of these correlations, pupillometry has been used as an indirect measure of brain state.

Changes in the behavior of an animal, the pupil dilation (mydriasis), and the brain state often coincide on the scale of seconds. It has been suggested that these changes are linked by the activities of neuromodulatory systems (Jones, 2004; Lee and Dan, 2012) and changes in the activity of noradrenergic and cholinergic circuitry correlate with changes in pupil dilation. Here we review evidence that changes in pupil dilation reflect and, in some instances, may be caused by the activity of neuromodulatory pathways.

## Mechanisms controlling pupil diameter and the effects of luminance

**Figure 1:**
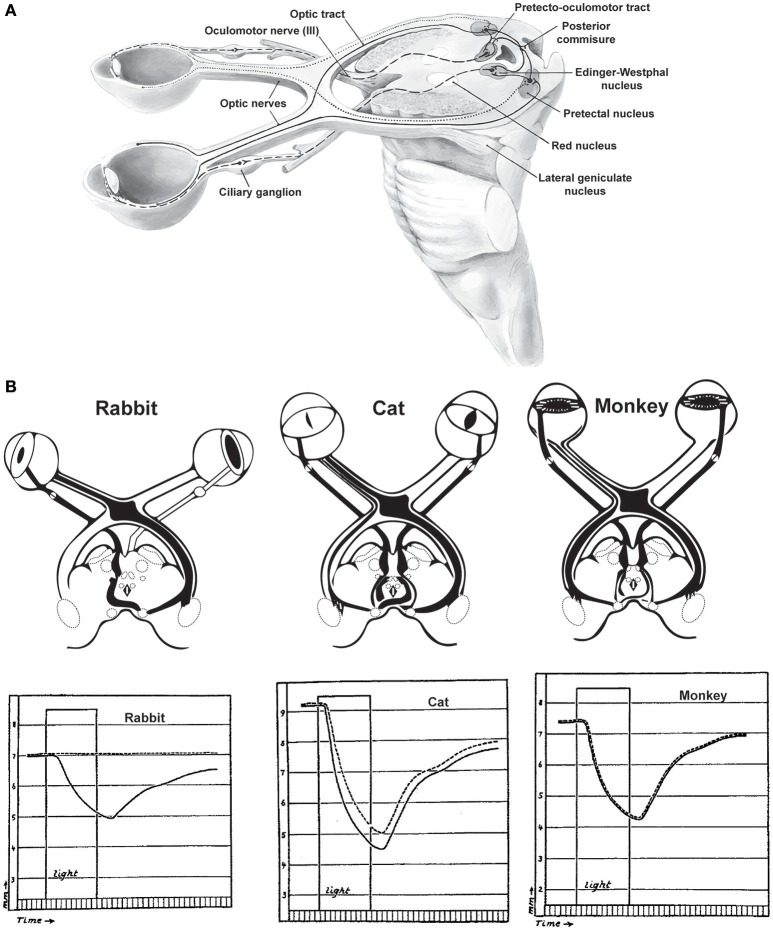
The pupillary light reflex. **(A)** Neural circuits involved in the pupillary light reflex. **(B)** Responses of various species to light stimulation of the left eye. In rabbits, which lack many decussating optic axonal fibers, unilateral light stimulation produces no consensual light reflex in the contralateral eye (dashed lines). In contrast, species which have more decussating than non-decussating fibers have either imperfect consensual reactions to unilateral light stimulation (cats) or symmetric direct consensual reactions (monkeys). Duration of the light stimulation is indicated by the rectangular “light” period in each plot. Reprinted with permission from Kardon (2005).

Pupil dilation is altered by luminance in the visual environment as well as during arousal, defined here as periods of heightened sensory responsiveness and perception that involve autonomic and endocrine activation. The diameter of the pupil is controlled by the iris sphincter muscle that constricts the pupil and the dilatory pupillary muscle that promotes pupil dilation (Borgdorff, 1975; Levin and Kaufman, 2011). The iris sphincter muscle is stronger than the dilatory pupillary muscle, making the iris sphincter muscle the primary controller of pupil diameter. The iris sphincter muscle is controlled via cholinergic preganglionic motoneurons in the Edinger–Westphal (E–W) nucleus, which project to the ciliary ganglion of the third cranial nerve from which the iris sphincter muscle is controlled via the ciliary nerve (Beatty and Lucero-Wagoner, 2000). Activity of projecting neurons in the E-W nucleus drives contraction of the iris sphincter muscle and constriction of the pupil; inhibition of E-W neurons relaxes the iris sphincter muscle, permitting dilation (Figure 1A).

The iris sphincter and dilatory pupil muscles are under the control of the parasympathetic and sympathetic nervous systems, respectively. Sympathetic nervous activity promotes dilation and parasympathetic activity promotes constriction of the pupil, but changes in pupil diameter driven by changes in luminance and arousal engage sympathetic and parasympathetic nervous systems differentially, with lesions in sympathetic pathways primarily impeding dilation in response to changes in arousal, but not luminance (Loewenfeld, 1958). Lesions in parasympathetic pathways primarily affect the pupillary light response.

The diameter of the pupil is modulated by changes in luminance, with dilation of the pupil in low light conditions and constriction in bright light, the latter referred to as the pupillary light response. The pupillary light response is thought to optimize retinal illumination and, thereby, visual perception. Two retinal mechanisms are thought to be involved in detecting the changes in luminance that control pupil diameter. In primates, transient changes in luminance are reflected in the activity of rod photoreceptors and are relayed to melanopsin-expressing retinal ganglion cells that project to the pretectal olivary nucleus (Clarke and Ikeda, 1985; Gamlin et al., 1995). Cells of the pretectal olivary nucleus drive pupil constriction via projections onto cholinergic preganglionic motoneurons in the E–W nucleus (Beatty and Lucero-Wagoner, 2000; May et al., 2008). Rods adapt under sustained illumination, under which conditions pupil diameter is controlled by the melanopsin-expressing retinal ganglion cells, which are intrinsically photosensitive and display little adaptation (Keenan et al., 2016). In mice, transient changes in luminance may be detected directly by melanopsin-expressing cells in the iris sphincter muscle to drive pupil constriction, instead of through the rod photoreceptor pathway (Wang et al., 2017).

Luminance can also affect the diameter of the contralateral pupil, but the coupling of pupil diameters is species-specific. In species with significant overlap of visual fields such as primates, increases in luminance at a single eye produce almost equal changes in the diameters of both pupils (Kardon, 2005). In species with almost complete decussation of visual and pupillary input, such as rabbits, illumination in a single eye produces almost no change in pupil diameter in the contralateral eye (Figure 1B). In species with intermediate binocularity, such as cats, luminance exerts intermediate modulation of the contralateral pupil diameter (Loewenfeld, 1958). While less characterized, rodents generally display intermediate binocularity, leading us to infer that pupil diameter is likely less coupled across eyes in rodents than has been observed in cats or primates.

The pupil can also undergo periods of spontaneous, oscillating constrictions, and dilations that occur independently of changes in illumination or of eye movements. These changes are termed pupillary “hippus” and are thought to result primarily from parasympathetic nervous system activity (Turnbull et al., 2017). Pupillary hippus is reduced in periods of wakefulness and, like dilation, may be a useful measurement of behavioral state (Lüdtke et al., 1998; Prasad et al., 2011). However, further studies are needed to fully understand the relationship between hippus and brain states.

## Noradrenergic control of pupil size?

Changes in the state of the cortex and the pupil may be coupled by alterations in neuromodulatory signaling, particularly from the noradrenergic system. Noradrenergic neurons of the locus coeruleus are activated in response to sleep-wake transitions (Aston-Jones and Bloom, 1981; Carter et al., 2010), noxious stimuli (Rasmussen et al., 1986; Hirata and Aston-Jones, 1994), novel sensory stimulation (Hervé-Minvielle and Sara, 1995), and shifts in perception (Aston-Jones and Cohen, 2005). Intriguingly, many of these same stimuli are associated with changes in pupil diameter (Ellermeier and Westphal, 1995; Einhäuser et al., 2008; Kloosterman et al., 2015). Based on these findings, changes in the pupil diameter have been proposed to directly correlate with changes in the activity of locus coeruleus.

If locus coeruleus is causally responsible for pupil dilation, activity of locus coeruleus projection neurons should correlate with and precede pupil dilation. In mice, activity in noradrenergic projections to the cortex is correlated with increases in pupil diameter, even in the absence of locomotion (Reimer et al., 2016) and in extracellular recordings from locus coeruleus in rhesus macaques, spiking of locus coeruleus units tends to precede increases in pupil diameter by a mean of 335 ms (Joshi et al., 2016). In humans, studies combining high-resolution fMRI and pupillometry have also shown that blood-oxygen level dependent (BOLD) activity in brainstem areas overlapping with locus coeruleus increases in concert with pupil diameter in resting conditions and surrounding choices made during behavioral tasks (Murphy et al., 2014; de Gee et al., 2017).

The E–W nucleus may receive projections from locus coeruleus (Breen et al., 1983), with noradrenaline acting on neurons in the E-W nucleus through inhibitory α2-adrenergic receptors (Koss, 1986), but a direct projection from locus coeruleus to the E–W nucleus is controversial (Nieuwenhuis et al., 2011). Others have proposed that the correlation of locus coeruleus activity and pupil dilation possibly resulting from a common presynaptic input to locus coeruleus and the E–W nucleus (Nieuwenhuis et al., 2011). According to the indirect model, the paragigantocellularis nucleus (PGi) of the ventral medulla provides this common input, influencing pupil diameter via the E–W nucleus and co-activating the noradrenergic system. The superior colliculus also receives presynaptic input from the locus coeruleus and projects to the E–W nucleus where it may influence pupil dilation (Wang and Munoz, 2015). In support of a contribution from this circuit, spiking in monkey superior colliculus neurons often precedes pupillary changes (Joshi et al., 2016).

Electrical stimulation of locus coeruleus in anesthetized rodents and awake monkeys evokes pupil dilation, and this has been interpreted as evidence for direct coupling of locus coeruleus to pupil diameter (Joshi et al., 2016; Reimer et al., 2016). However, pupil dilation following electrical stimulation of the locus coeruleus happens on a relatively slow time-scale (~500 ms in monkeys, 1 s in mice), suggesting an indirect pathway may be involved. Additionally, pupil dilation during locus coeruleus stimulation might result from antidromic stimulation of projections into locus coeruleus from PGi or superior colliculus. Future manipulations of activity in only noradrenergic locus coeruleus neurons are necessary to determine whether noradrenergic neurons directly influence pupil size.

## Other neuromodulatory systems co-active with pupil dilation

Like noradrenergic neurons, cholinergic neurons of the basal forebrain are active during pupil dilation (Nelson and Mooney, 2016), sleep-wake transitions (Xu et al., 2015), periods of whisking in rodents (Eggermann et al., 2014), and following mildly aversive punishments such as an air puff (Hangya et al., 2015). Similarly, the activity of cholinergic axons, measured with calcium indicators expressed in cholinergic axons in cortex, increases with pupil dilation and is reduced during constriction (Nelson and Mooney, 2016; Reimer et al., 2016). Since pupil dilation correlates with the activity of both cholinergic and noradrenergic cells, pupil size is likely an unreliable measure of the activity of either neuromodulator.

**Figure 2:**
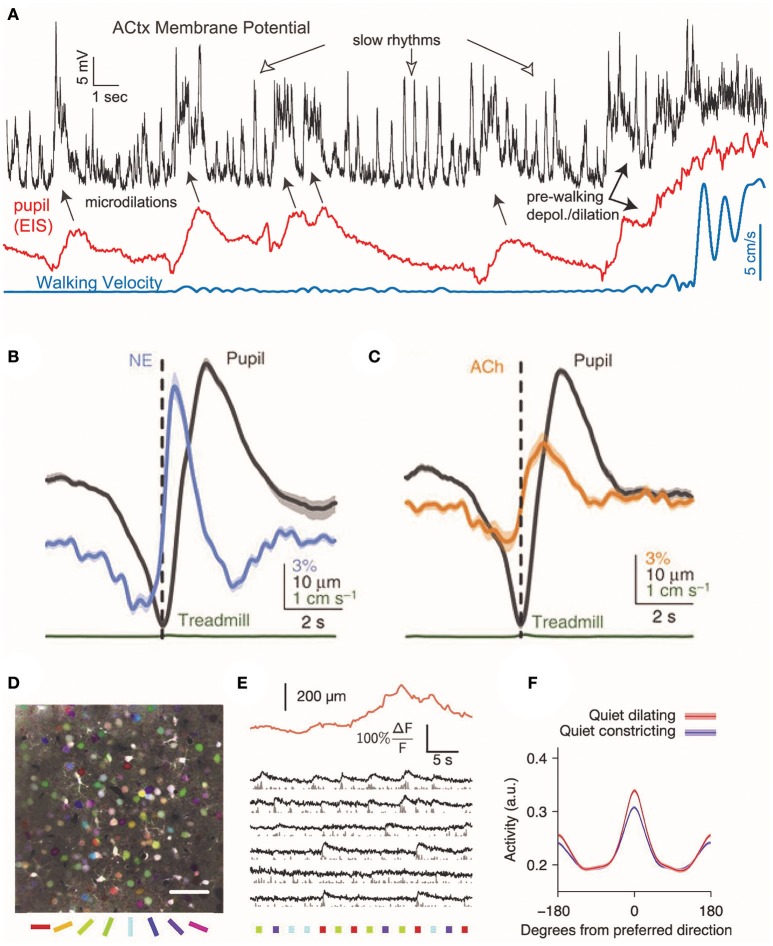
Cortical responses during pupil dilations. Changes in cortical pyramidal cell membrane potential coincide with changes in neuromodulatory signaling in the mouse auditory cortex. **(A)** During rapid, brief dilations (microdilations) in the absence of locomotion (blue), the cortical pyramidal cell membrane is depolarized 5–20 mV (black). Periods of locomotion are accompanied by membrane depolarization and large pupil diameters (red: eye-indexed state (EIS) is a measure of infrared reflectance used as a proxy for pupil diameter). Reprinted with permission from McGinley et al. (2015). **(B,C)** In the absence of locomotion, the activity of cholinergic and noradrenergic axons increases before pupil dilation. The activity of noradrenaline axons precedes cholinergic axons before pupil dilation. Reprinted with permission under the Creative Commons License from Reimer et al. (2016). **(D)** Temporal mean projection from layer 2/3 of mouse visual cortex showing neurons labeled with Oregon Green BAPTA. Somata are pseudo-colored with their preferred orientation. **(E)** Example responses of layer 2/3 somata to presentations of oriented stimuli (colored bars below image) with simultaneous measurement of pupil size (orange trace). **(F)** During periods without locomotion, pupil dilation is associated with an increase in the mean response to preferred direction, resulting in increased orientation selectivity. Reprinted with permission from Reimer et al. (2014).

Noradrenergic neurons from locus coeruleus send axons to the basal forebrain and can depolarize cholinergic neurons via α1 adrenergic receptors (Jones, 2004), raising the possibility that noradrenergic activity drive cholinergic activation. Consistent with this suggestion, noradrenergic axon activity begins ~1 sprior to the peak of pupil dilation and cholinergic axon activity ~0.5 s later (Figures 2B,C) (Reimer et al., 2016).

Pharmaceutical agents that act on neuromodulatory systems can also produce changes in pupil dilation and might suggest the involvement of these neurotransmitters in pupillary changes (Faber, 2017). For example, the serotonergic agonist lysergic acid diethylamide (LSD) and antagonist metergoline produce pupil dilation and constriction, respectively, when administered to humans (Vitiello et al., 1997; Schmid et al., 2015). It has been suggested that serotonergic effects on pupil diameter might result from interactions with the locus coeruleus (Yu et al., 2004; Einhäuser et al., 2008).

Systemic administration of muscarinic acetylcholine antagonists such as scopolamine produce pupil dilation (Vitiello et al., 1997). However, systemically-administered cholinergic drugs could also act directly at the iris sphincter and dilatory pupil muscles by influencing cholinergic neuromuscular junctions. In clinical settings cholinergic antagonists are routinely applied directly to the eye to dilate the pupil, with no likely involvement of the CNS (Duvall and Kershner, 2006). These pharmaceuticals have tonic effects on pupil, making it difficult to infer from these effects the timescale over which the neuromodulatory systems influence the pupil.

The highly-interconnected nature of brain circuits and the plethora of effects of neuromodulatory drugs make it difficult to identify the routes of action in many pharmacology experiments and determine the direct effects of each neuromodulatory system on pupil diameter, but the literature is generally supportive of the idea that changes in brain state involving neuromodulators, including those induced pharmacologically, may affect pupil diameter.

## Behavioral states coinciding with pupil dilation

Changes in pupil diameter have long been hypothesized to correlate with changes in brain state (Schiff, 1875). Early human studies suggested pupil changes were linked with gender-specific interest in the content of a picture and with the mental activity required to solve a complex arithmetic problem, with more difficult multiplication problems being associated with more pupil dilation (Hess and Polt, 1960, 1964). Later findings demonstrated that pupil diameter correlates with heart rate and skin conductance, measures of autonomic nervous system activity, when viewing emotionally salient images (Bradley et al., 2008). Pupil dilations are also linked to perceptual switches when human subjects are presented with an unchanging object that can be perceived multiple ways (Einhäuser et al., 2008). This finding and others have led to the suggestion that pupil dilation reflects uncertainty, surprise, and reflects reward prediction errors (Preuschoff et al., 2011; Lavín et al., 2013).

Changes in pupil diameter predict of behavioral outcomes in a variety of tasks. In predictive-inference tasks, pupil dilations are correlated with dynamic changes in the learning rate of subjects (Nassar et al., 2012). Given that pupil dilations coincide with alterations in neuromodulatory signaling, these effects on learning may be mediated by neuromodulatory receptors altering the threshold for inducing synaptic plasticity (Larsen et al., 2010). Pupil changes are also associated with periods of uncertainty and mental effort (Alnæs et al., 2014). For example, the pupil dilates during a protracted decision in humans and is correlated with a decision that contradicts a previously-held bias (de Gee et al., 2014). Additionally, in studies of humans performing a gambling task, pupil diameter was greater in trials where subjects explored slot machines with a lower expected payout instead of choosing the slot machine with the highest estimated payout (Jepma and Nieuwenhuis, 2011). These results suggest pupillary responses may reflect surprise and adaptations to environmental volatility. Interestingly, individuals scoring high in anxious trait measurements show a reduced pupillary response to volatile periods of a behavioral task in which choices produce variable outcomes (Browning et al., 2015). Collectively, these results suggest that pupil diameter may reflect the cognitive activity that underlies behavioral choice during challenging tasks (Aston-Jones and Cohen, 2005) and may be altered in psychiatric disorders.

Changes in locomotion, brain states and pupil size are correlated in time (Reimer et al., 2014), making it difficult to determine whether pupil dilation is driven by a single or multiple neuromodulatory circuits and, if multiple, whether the circuits that relate pupil size and locomotion, and pupil size and brain states act independently. These possibilities can be disentangled by examining changes in pupil diameter during quiet wakefulness, in which running and exploratory behaviors do not occur but small, rapid changes in pupil dilation persist. Under these conditions, dilations are correlated with desynchronization of the membrane potential in visual and somatosensory cortices (Reimer et al., 2014). The correlation between changes in pupil diameter and cortical membrane potential synchrony during quiet wakefulness persists in the FVB strain of mice, in which retinal ganglion cells degenerate in adulthood (Reimer et al., 2014), suggesting that changes in cortical state during pupil dilations can occur independently of both visual input and active exploratory states. Overall, these results suggest that changes in brain state, and related changes in pupil diameter, occur in the absence of overt shifts in locomotion or behavior.

## Changes in cortical state during pupil dilations

Alterations in pupil diameter are coupled to changes in the state of the neocortex, which is reflected in the membrane potential of pyramidal neurons (Destexhe et al., 2003). Pupil dilation typically correlates with a behavioral shift to a more active state. In mice, pupil dilation is associated with periods of whisking and increases in locomotion (Reimer et al., 2014; McGinley et al., 2015). In the period preceding walking, the cortical membrane potential typically becomes depolarized, followed about 1 s later by dilation of the pupil and 3–4 s later by walking (McGinley et al., 2015). Larger pupil diameters in rodents are observed during periods preceding locomotion than during the quiet wakefulness; however, smaller, brief microdilations are observed even in the absence of locomotion (McGinley et al., 2015; Vinck et al., 2015). Transient in nature, microdilations may be similar to task-invoked pupillary responses measured in humans (Hess and Polt, 1964), which are thought to reflect cognitive demands of a task (Beatty, 1982).

In mice, prolonged pupillary constriction is associated with slow rhythmic oscillations in the membrane potentials of pyramidal neurons in auditory cortex at frequencies below 10 Hz (Figure 2A) (McGinley et al., 2015). During small, lasting dilations, pyramidal cell membrane potential became hyperpolarized and low frequency oscillations were suppressed. In contrast, larger dilations were associated with 50–100 Hz membrane potential oscillations and depolarization. In mouse auditory cortex, brief microdilations in the absence of walking were also associated with 5–20 mV membrane depolarizations of deep layer pyramidal cells (McGinley et al., 2015), although interestingly this effect was not observed in supragranular pyramidal neurons in visual cortex (Reimer et al., 2014). In an auditory go/no-go behavioral task, performance was highest during intermediate pupil diameters, when pyramidal cells in auditory cortex were hyperpolarized for ~100 ms prior to the stimulus (McGinley et al., 2015). This suggests that optimal behavioral performance may be at intermediate arousal levels when the pupil is dilated to 40–60% of maximum.

What consequences might changes in brain state that correlate with pupil diameter have on information processing? In mouse visual cortex, neuronal calcium transient responses to the preferred orientation of drifting gratings are increased during pupil dilation, resulting in increased orientation selectivity during quiet wakefulness (Reimer et al., 2014). Visual cortical responses to frames of a presented movie also become more reliable and selective during periods in which the pupil is undergoing fast pupil dilations, indicating that pupil dilation correlates with an increase in the fidelity of preferred stimuli (Reimer et al., 2014). Changes in pupil diameter also correlate with the signal-to-noise ratio of a neuronal response to visual stimuli: when mice are delivered an air puff to produce an increase in arousal and the corresponding dilation of the pupil, the average signal-to-noise ratio is higher (Vinck et al., 2015). These effects on visual encoding are similar to that observed during increased locomotion (Dadarlat and Stryker, 2017), but are observed in its absence. This result further demonstrates that pupil dilation correlates with movement-independent changes in internal brain state that enhance sensory encoding. Enhanced sensory coding with pupil dilation is similar to findings that stimulation of noradrenergic or cholinergic neurons can increase cortical neuron responsiveness and the signal-to-noise ratio of responses to sensory stimuli (Martins and Froemke, 2015; Minces et al., 2017). It is possible that changes in pupil diameter merely coincide with alterations in sensory processing rather than driving them, but these findings suggest that changes in neuromodulation mechanistically link these two processes.

## Microcircuits underlying pupil-linked changes in cortical state

As discussed above, multiple neuromodulatory systems may participate in the changes in cortical state that coincide with pupil dilation. However, there is increasing consensus on one cortical circuit that may strongly contribute to the cortical membrane depolarizations observed during active behavioral states and with pupil dilation (Stryker, 2014). In mouse visual, somatosensory, and prefrontal cortices, vasoactive intestinal polypeptide-positive (VIP+) interneurons form synapses onto somatostatin-positive (SST+) interneurons, which in turn inhibit glutamatergic pyramidal neurons (Lee et al., 2013; Pfeffer et al., 2013; Pi et al., 2013). Increases in the firing of VIP+ interneurons thereby depolarize pyramidal neurons and this coupling is observed during active states that correlate with pupil dilation. VIP+ interneurons express nicotinic acetylcholine receptors (Fu et al., 2014), receive input from noradrenergic axons (Paspalas and Papadopoulos, 1999) and are members of a larger class of interneurons that express the ionotropic receptor subtype 5HT3A (Lee et al., 2010; Pfeffer et al., 2013). Likely multiple neuromodulatory circuits directly influence cortical VIP+ interneurons. A parsimonious mechanistic explanation for the changes in cortical LFP and pyramidal cell membrane potential that correlate with pupil dilation is that the changes in behavioral state result in increases in neuromodulatory activity that depolarize cortical VIP+ interneurons while simultaneously modulating circuits involved in pupil dilation. In support of this explanation, cortical VIP+ neurons are depolarized during pupil dilation, while SST+ interneurons are hyperpolarized (Reimer et al., 2014).

Direct activation of VIP+ interneurons is likely only one of several mechanisms by which neuromodulators affect cortical states that coincide with changes in pupil diameter. Many of the neuromodulatory receptors expressed by VIP+ interneurons are also expressed by cortical pyramidal neurons, which also receive neuromodulatory input (Santana et al., 2004; Hedrick and Waters, 2015; Salgado et al., 2016). Additionally, increases in neuromodulatory tone during locomotion may increase the activity of thalamic projections to cortex independent of neuromodulator release into neocortex (Erisken et al., 2014; Roth et al., 2016). Importantly, however, fluctuations in pyramidal cell membrane potential during active exploratory behaviors coincident with cholinergic neuromodulation are preserved when thalamic activity is silenced (Eggermann et al., 2014), suggesting that acetylcholine acts in cortex, rather than indirectly through thalamus. Further studies are warranted to dissect how multiple pathways converge to influence the cortical state during periods of active behavior and pupil dilation.

## Conclusions

Pupillometry is a useful proxy for measuring transitions of the neocortex into active states. Changes in pupil diameter are primarily driven by luminance and the visual environment, necessitating careful separation of the effectors when seeking to use pupil diameter as a proxy for an internal brain state. Furthermore, an array of brain areas may be co-activated during pupil dilatory events and correlations between pupil diameter, multiple neuromodulatory systems and transitions to active to behavioral states make it difficult to use changes in pupil diameter as a readout for the activity of a single neuronal circuit (Figure 2). Therefore, future studies that further temporarily dissect the involvement of each neuromodulatory circuit during changes in pupil diameter are still needed.

The time courses of the activation of neuromodulatory systems during pupil dilation and more broadly during increases in behavioral activity are just beginning to be understood in rodents (Reimer et al., 2014). How changes in pupil diameter in rodents relate to cognitive-driven changes in humans, and the similarities and differences in the respective mechanisms, also remains to be determined.

## Author contributions

All authors listed have made a substantial, direct and intellectual contribution to the work, and approved it for publication.

### Conflict of interest statement

The authors declare that the research was conducted in the absence of any commercial or financial relationships that could be construed as a potential conflict of interest.
